# Development of a behavior change intervention to improve physical activity adherence in individuals with metabolic syndrome using the behavior change wheel

**DOI:** 10.1186/s12889-022-14129-1

**Published:** 2022-09-14

**Authors:** Dandan Chen, Hui Zhang, Nianqi Cui, Feng Song, Leiwen Tang, Jing Shao, Jingjie Wu, Pingping Guo, Na Liu, Xiyi Wang, Zhihong Ye

**Affiliations:** 1 Nursing Department, Affiliated Sir Run Run Shaw Hospital, Zhejiang University School of Medicine, No.3 East Qingchun Road, Shangcheng district, Hangzhou, 310020 China; 2 Department of Cardiology, Guizhou Provincial People’s Hospital, 83 East Zhongshan Road, Guiyang, 550002 No China; 3 Nursing Department, The Second Affiliated Hospital of Zhejiang University School of Medicine, No.88 Jiefang Road, Shangcheng district, Hangzhou, 310009 China; 4 Faculty of Medicine, National University of Malaysia, Kuala Lumpur, Malaysia; 5 Faculty of Medicine, Yunnan College of Business Management, No. 296 Haitun Road, Wuhua district, Kunming, 650106 China; 6 School of Nursing, Zhejiang University School of Medicine, No. 866 Yuhangtang Road, Xihu district, Hangzhou, 310012 China; 7 Women’s Hospital, Zhejiang University School of Medicine, No.1 Xueshi Road, Shangcheng district, Hangzhou, 310006 China; 8 Nursing Department, Chongqing Medical University Affiliated Second Hospital, No. 76 Linjiang Road, Yuzhong district, Chongqing, China; 9 School of Nursing, Shanghai Jiao Tong University, Chongqing South Road Huangpu District, No. 227, Shanghai, China

**Keywords:** Metabolic syndrome, Physical activity adherence, Behavioral Change Wheel, Mobile health

## Abstract

**Background:**

Adherence to physical activity is inadequate in adults with metabolic syndrome. Adherence to physical activity recommendations is crucial and can result in improved health outcomes and reduced medical burdens. A comprehensive behavior change intervention, including identifying determinants of adherence to physical activity recommendations, intervention options, intervention content and implementation options, was imperative for enhancing physical activity adherence. The aim of the study is to develop an intervention to increase physical activity adherence among individuals with metabolic syndrome.

**Methods:**

The study followed the eight steps of the Behavior Change Wheel guide, including defining the problem in behavioral terms (Step 1), selecting target behavior (Step 2), specifying target behavior (Step 3), identifying what needs to change (Step 4), identifying intervention functions (Step 5), identifying policy categories (Step 6), identifying behavior change techniques (Step 7), and determining model of delivery (Step 8). The semi-structured, in-depth interviews were employed to identify the determinants of adherence to physical activity among twenty-eight individuals with metabolic syndrome based on capability, opportunity, motivation and behavior model. Next, the intervention functions and policy categories were chosen to address these determinants. Finally, behavior change techniques were selected to assist in the delivery of the intervention functions and be translated into intervention content.

**Results:**

Our study identified eighteen facilitators and fifteen barriers to physical activity adherence. It resulted in the selection of seven intervention functions and nineteen behavior change techniques for the intervention program. Then, the current study identified an app as the delivery mode. Finally, a behavioral change intervention was generated for individuals with metabolic syndrome to increase physical activity recommendation adherence.

**Conclusions:**

The Behavior Change Wheel provided a systematic approach to designing a behavior change intervention, which helped improve the health outcomes and reduce medical burdens and economic burdens among individuals with metabolic syndrome. The findings suggested that potential intervention should pay special attention to increasing knowledge in metabolic syndrome, imparting skills of physical activity, offering a supportive environment, and providing suggestions on regular physical activity using the appropriate behavior change techniques. A feasibility study will be undertaken to assess the acceptability and effectiveness of the intervention program in the future.

## Background

Metabolic syndrome is a worldwide medical and public health concern [1, 2]. It is a cluster of cardiovascular risk factors, not limited to increased waist circumference (WC), high systolic blood pressure (SBP) or diastolic blood pressure (DBP), high triglyceride (TG) levels, low high-density lipoprotein cholesterol (HDL-C), and elevated fasting blood glucose (FBG) [3]. Metabolic syndrome is increasing and is likely to reach epidemic proportions [4]. It has been estimated that global prevalence of metabolic syndrome is about 25% [5]. The prevalence of metabolic syndrome was 8.8% in 1991–1995, 29.3% in 2011–2015, and 31.1% in 2015–2017 in China [2, 6]. It is associated with negative outcomes, including a high risk with type 2 diabetes, cardiovascular disease, and all-cause mortality [7]. Additionally, the cost of the metabolic syndrome is in trillions, and forecasted to rise in the future [8]. In Germany, Spain and Italy, the health service cost of metabolic syndrome among patients with hypertension is €24,427, €1,900 and €4,877 million and expect to rise by 59%, 179% and 157% respectively by 2020 [9], which places heavy medical and economic burdens on individuals and the healthcare system. Thus, the management of metabolic syndrome is of paramount importance.

Physical activity has a substantial positive effect on metabolic syndrome [10]. Physical activity is defined as “any movement of the body produced by skeletal muscles that results in energy expenditure” [11]. It is imperative to sustain participation in physical activity since metabolic syndrome is a prevalent long-term condition, which requires substantial expenditure of effort and continuous perseverance [12]. However, during the process of participation, adherence to physical activity recommendations remains a great challenge among people with metabolic syndrome [13, 14]. In other words, the individuals' health-related behaviors (including taking medication, implementing lifestyle changes, etc.) are not completely consistent with the advice (prescriptions) provided by the health care providers [15]. Fappa et al., [16] found that individuals with metabolic syndrome may have poor metabolic syndrome parameters due to inadequate adherence to physical activity. Keller et al., [17] reported that adherence declined with an increase in the recommended frequency of exercise. Gallardo-Alfaro et al., [18] showed that the adherence to physical activity recommendations needed to be improved among people with metabolic syndrome. Chen et al., [19] reported that the physical activity level of individuals with metabolic syndrome was low. Therefore, it is essential to identify the facilitators and barriers to physical activity adherence and develop intervention strategies to improve it.

It has been found that most existing interventions to improve physical activity adherence have some effectiveness, but they tend to be poor in the application of theory [20–22], which may limit their success and lead to suboptimal adherence [19]. Theory-based intervention could enhance the effectiveness of behavior change components [23], as the relationships between constructs, that are predictive of behavior change, can be understood, translated into intervention content, and then examined for an explanation of how an intervention achieved, or did not, its desired outcome [24]. Thus, it is necessary to develop theoretically-informed intervention strategies to encourage persons to sustain physical activity and integrate them into their daily lives in China.

**Fig. 1:**
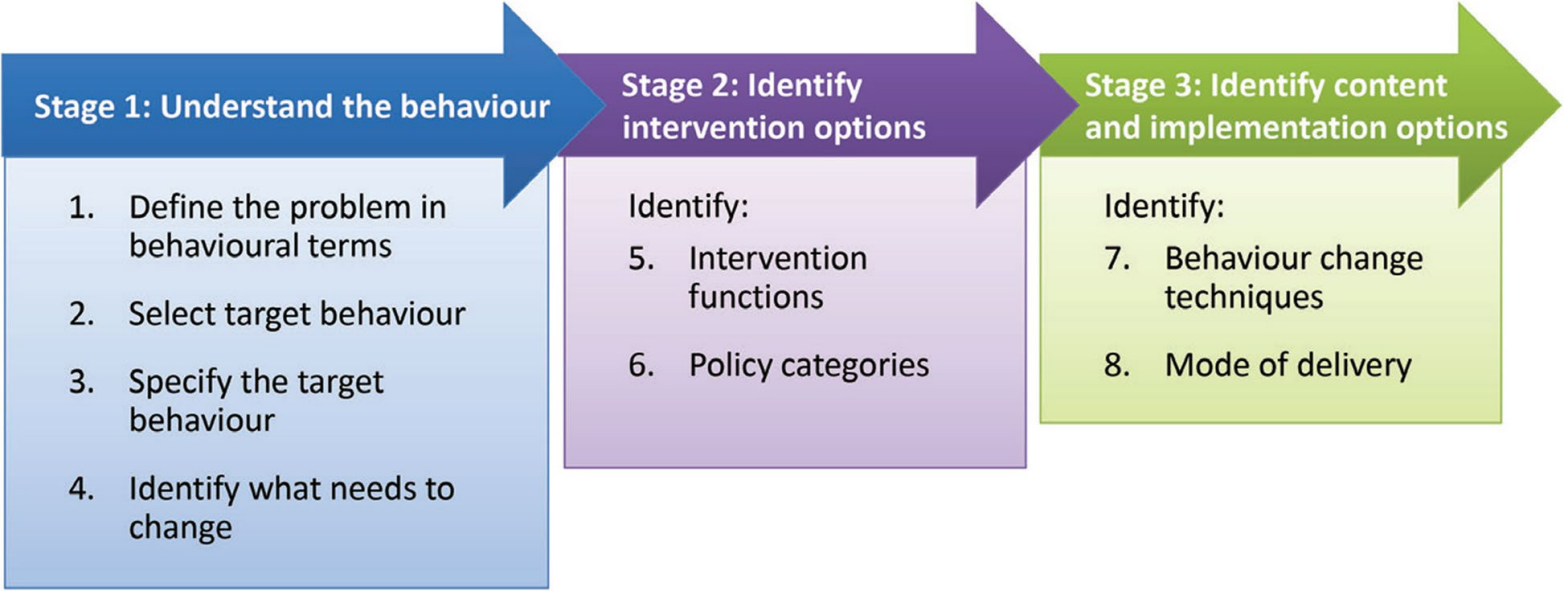
Stages involved in an intervention development using the BCW [27] (used with permission from authors)

**Fig. 2:**
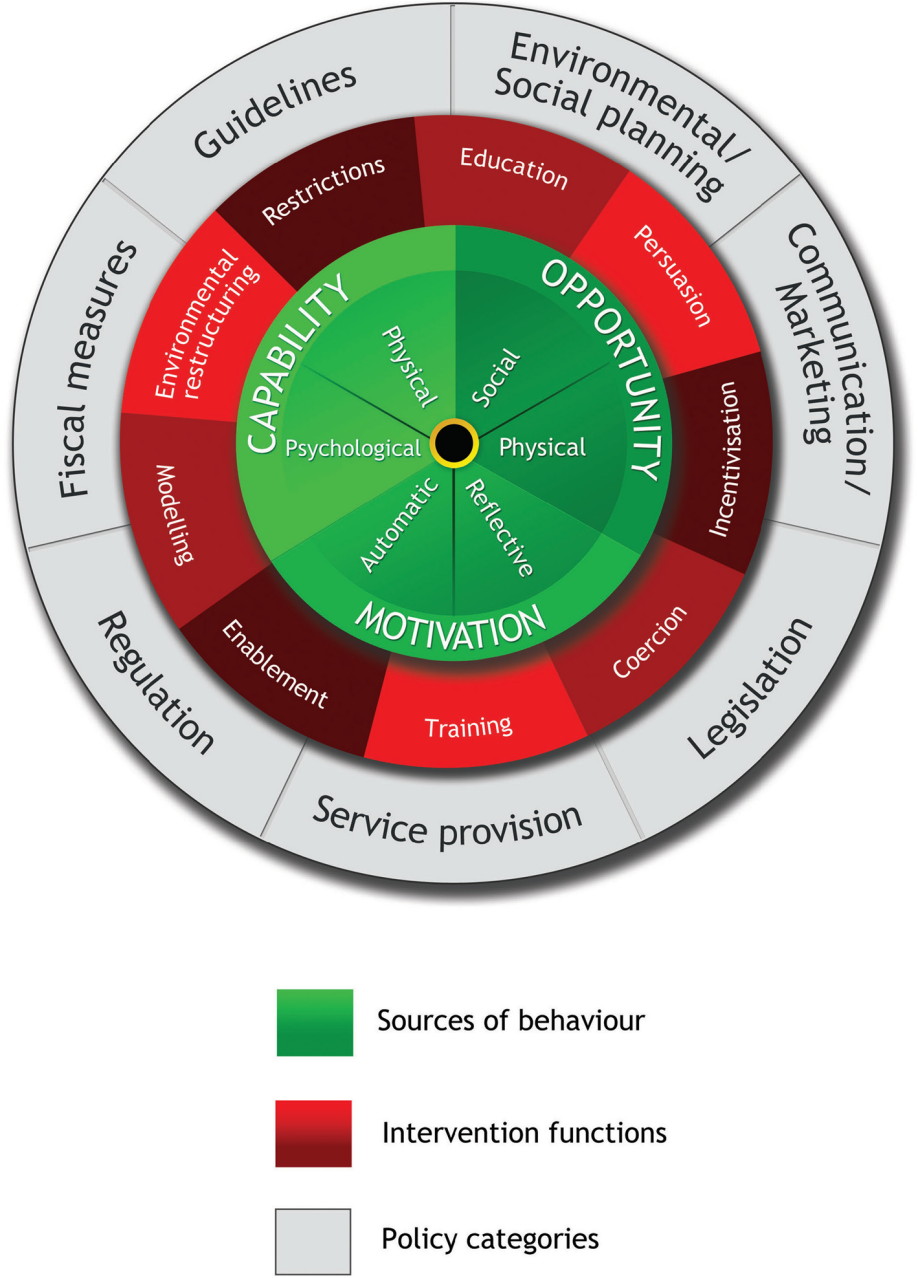
The Behavior Change Wheel (used with permission from authors)

Behavioral science frameworks provide theory to help determine the potential impacts that support or disrupt initiation and maintenance of behavior change [25]. The Medical Research Council’s (MRC) framework on developing and evaluating complex interventions aims to help researchers adopt suitable methodologies [26]. Firstly, the behavior change wheel (BCW) framework wa**s** selected to guide the intervention development process for its ability to address the broad scope and incoherent definitions of theoretical constructs identified within existing theoretical frameworks and provides a systematic and transparent method for promoting behavior change [27, 28]. The BCW synthesized 19 behavior change frameworks and provided a three-stage intervention method (see Fig. 1): understanding the behavior (Stage 1), identifying intervention options (Stage 2) and identifying content and implementation options (Stage 3). The first stage involved four steps to understand the behavior: defining the problem in behavioral terms (Step1), selecting target behavior (Step2), specifying target behavior (Step3), and determining what needs to change (Step4). The second involved two steps (Step5 and Step6): identifying intervention functions (Step5) and policy categories (Step6). The third stage included two steps: identifying behavior change techniques (BCTs) (Step7) and model of delivery (Step8) [27]. In short, it consisted of three layers (see Fig. 2). At its hub is the capability, opportunity, motivation, and behavior (COM-B) model, which focuses on exploring determinants of target behavior [27]. Capability can be either ‘physical’ (having the physical skills, strength, or stamina) aspects required to perform the behaviour or ‘psychological’ (having the knowledge, psychological skills, strength, or stamina) aspects required to perform the behaviour. Opportunity can be ‘physical’ (what the environment allows or facilitates in terms of time, triggers, resources, locations, physical barriers, etc.) or ‘social’ (including interpersonal influences, social cues, and cultural norms). Motivation may be ‘reflective’ (involving self-conscious planning and evaluations) or ‘automatic’ (involving wants and needs, desires, impulses, and reflex responses). Additionally, the Theoretical Domains Framework (TDF) [29] has been added to the BCW to further unpack factors identified in the COM-B model into 14 theoretical domains. The second layer of the BCW is nine intervention functions, through which an intervention could modify behavior [27]. The third layer is seven policy categories, as high-level strategies, which help support the implementation of intervention functions [27]. Moreover, the BCTs from the version 1 of the BCT taxonomy (BCTTv1) are active ingredients and have been linked to the BCW to assist in delivery of intervention functions [30]. Additionally, the BCW provides theory-based linkages between COM-B components, intervention functions, BCTs, and policy categories [27]. The BCW has been widely applied to design behavior change interventions that target some health-related behaviors, such as eating habits [31], sedentary behavior [32], weight management [33] and physical activity behavior [34, 35]. Moreover, the intervention drawing on the BCW framework has showed benefits in improving the adherence to healthy eating, exercise, and body composition [36]. However, no known research has attempted to understand the physical activity behavior among individuals with metabolic syndrome using the BCW framework in China.

Therefore, the aim of this study was to systematically develop a comprehensive behavior change intervention to support adherence to physical activity in people with metabolic syndrome in China guided by the BCW framework. We hope that this study can be used by health care professionals when they plan to provide physical activity guidance for their clients, and ultimately improve the clinical health outcomes and quality of life of people with metabolic syndrome. Additionally, the methodology identified in the current study could provide references for other researchers developing behavior change interventions.

## Methods

Based on the BCW framework, we developed a three-stage intervention that included eight steps [27].

### Stage 1: Understanding the behavior

#### Step 1: Define the problem in behavioral terms

This step required researchers to formulate the problem in behavioral terms and assess two aspects: (i) who is involved in performing the behavior and (ii) what the behavior is [27]. Evidence on physical activity adherence was reviewed to identify the problem among people with metabolic syndrome. We searched Cochrane Library, Embase, Web of Science, PubMed, CINAHL, Chinese National Knowledge Infrastructure, Weipu and Wanfang for papers published through March 2021 using the following keywords: “metabolic syndrome”, “physical activity”, “exercise”, “movement”, “physical therapy”, “strength training”, “aerobic training”, “resistance training”, “weight training”, “physiotherapy”, “stretching exercise”, “kinesiotherapy” and “lifestyle”. Manually searching relevant papers for cited references was also conducted if necessary.

#### Step 2: Select target behavior

Step 2 involved determining the target behaviors that might solve the defined problems in Step 1. The final target behavior was selected based on four criteria from the BCW framework: (i) how much of an impact changing the behavior will have on the desired outcome, (ii) how likely it is that the behavior can be changed, (iii) how likely it is that the behavior will have a positive or negative impact on other, related behaviors, and (iv) how easy it will be to measure the behavior [27]. We carried out a literature search on physical activity management measures among people with metabolic syndrome to select potential target behaviors.

#### Step 3: Specify target behavior

The BCW guided us to specify the target behavior through six questions, including (i) who needs to perform the target behavior, (ii) what they need to do differently to achieve change it, (iii) where and (iv) when they do it, (v) how often, and (vi) with whom they do it [27]. To specify the target behavior, we reviewed existing literature on physical activity interventions for individuals with metabolic syndrome.

#### Step 4: What needs to change?

We performed a qualitative, descriptive study with a constructionist epistemology [37] that acknowledges that knowledge is constructed based on perception and experiences of individuals, and constructed via speech to understand the world [38] to explore determinants of physical activity adherence in this step, which included both barriers and facilitators. These determinants were then mapped into COM-B components and TDF domains. The study design was conducted following the Standards for Reporting Qualitative Research (SRQR) [39]. Three domains are included in the COM-B model: capability, opportunity, and motivation, which interact with one another to enable a behavior to occur. The TDF includes fourteen domains that can be condensed to fit the three constructions of the COM-B model, as follows: capability (knowledge, cognitive and interpersonal skills, memory, attention and decision processes, behavioral regulation, and physical skills), opportunity (social influences, environmental context, and resources), and motivation (reinforcement, optimism, emotions, social/professional role and identity, beliefs about capabilities, beliefs about consequences, goals, and intentions) [40].

##### ***Participants and settings***

Individuals who have been diagnosed with metabolic syndrome according to the criteria proposed by the 2009 Joint Scientific Statement (harmonizing criteria) [3] and aged over 18 years were recruited. People who had severe diseases and could not communicate effectively due to oral diseases were excluded. Between May and August, 2021, two researchers recruited participants by distributing a recruitment advertisement. If individuals agreed to participate, they were given information about the study, and then they were asked to fill out a written informed consent form. We recruited participants with rich information through a purposive and criterion-based sampling method. Participants who met the criteria were selected by considering their representativeness of gender, age, education level, residence, income, and occupation to obtain rich information. Researchers conducted the study at a health promotion center of a general university hospital in Hangzhou, China.

##### ***Data collection***

From May to August 2021, we conducted semi-structured, one-on-one interviews. An interview guideline (see Table 1) was developed based on the COM-B model and the TDF domains. The first author (CDD) interviewed people with metabolic syndrome in a quiet room. We recorded all interviews with the participants’ consent. The time of interviews ranged from 23 ~ 78 min. Data collection and analysis were conducted simultaneously. Researchers (CNQ and ZH) transcribed verbatim audio materials in Chinese, and researchers (CDD and ZH) coded the interviews within 24 h. Then, the next participant was interviewed. When there were no new themes occurring that meant thematic saturation, data collection could be stopped [41]. Additionally, we interviewed 3 additional people with metabolic syndrome to confirm that no new themes appeared.

**Table 1:** Interview schedule

COM-B	TDF	Question
Psychological capability	Knowledge	How do you understand metabolic syndrome and its physical activity measures?
	Behavior regulation	How do you ensure that your physical activity is regular? Are there procedures or ways that encourage you to perform regular physical activity?
	Memory, attention and decision process	How do you decide whether or not to perform regular physical activity? Do you use any prompts?
Physical capability	Skills	When it comes to physical activity, what skills do you think are necessary?
Social opportunity	Social influences	How do your parents or friends or other people help or hinder you perform regular physical activity?
Physical opportunity	Environmental context and resources	What factors of work or/and home environment support or hinder your maintenance of regular physical activity?
Reflective motivation	Social/professional role and identity	To what extent do you see maintaining regular physical activity as part of your role?
	Belief about capabilities	What is your level of confidence about maintaining regular physical activity?
	Beliefs about consequences	What do you think will happen if you do regular physical activity?
	Optimism	How confident are you that maintaining regular physical activity will have a positive outcome?
		How confident are you that you can overcome the barriers you face to maintain regular physical activity?
	Intention	Do you intend to maintain regular physical activity? (Prompt: If not, why not?)
	Goals	What are your goals when you maintain regular physical activity?
Automatic motivation	Reinforcement	What would be an incentive to maintain regular physical activity?
	Emotion	Discuss how you think maintaining regular physical activity would make you feel? Does it give you any particular feelings or emotions?

##### ***Data analysis***

The transcribed interview sessions were analyzed by the coders using a thematic analysis [42]. CDD and ZH independently read and reread the transcripts and interview notes to code inductively and then produce themes. A continuous analysis of the data and frequent discussions among the authors were done to refine and define the themes and subthemes. Two researchers categorized the specific themes into the most relevant domains (COM-B elements and TDF domains). Differences were discussed with the research team until a consensus was reached during inductive coding and deductive categorizing. When analyzing data, we wrote a reflective note to remain calm and objective and thus reduce the impact of any pre-existing notions. Additionally, in order to ensure trustworthiness, we enhanced the credibility, transferability, dependability and confirmability of the present study [43]. Credibility was ensured by our research team who discussed any differences in methodological issues and data analysis. Regarding transferability, this article described the participants’ characteristics, contexts and verbatim quotes to enable the reader to make judgments about the generalizability of the results. Dependability and confirmability were achieved by cross-checking transcripts by people who did not participate in the transcription process.

### Stage 2: Identifying intervention options

#### Step 5: Identifying intervention functions

According to the BCW, the COM-B domains and TDF were connected to the intervention functions [27, 44]. The intervention functions included education, training, restriction, persuasion, incentivization, coercion, modeling, environmental restructuring, and enablement [27, 44]. Education refers to increasing understanding and knowledge of targeted behaviors [27]. Persuasion refers to using communication to induce positive or negative feelings or stimulate action [27]. Incentivization refers to creating an expectation of reward [27]. Coercion refers to creating an expectation of punishment or cost [27]. Training refers to imparting skills [27]. Restriction refers to using rules to reduce the opportunity to engage in the target behavior (or to increase the target behavior by reducing the opportunity to engage in competing behaviors) [27]. Environmental restructuring refers to changing the physical or social context [27]. Modelling refers to providing an example for people to aspire to or imitate [27]. Enablement refers to increasing means/reducing barriers to increase capability or opportunity [27]. According to BCW, intervention functions were selected on the basis of their affordability, practicability, effectiveness, acceptability, side-effects and safety, and equity (APEASE) [27]. Affordability refers to whether the cost of the proposed intervention is within budget [27]. Practicality refers to the extent to which an intervention is delivered as designed through the means intended to the target population [27]. Effectiveness refers to the effect size of the intervention in relation to the desired objectives in a real world context [27]. Cost-effectiveness refers to the ratio of effect (in a way that has to be defined, and taking account of differences in timescale between intervention delivery and intervention effect) to cost [27]. Acceptability refers to the extent to which an intervention is judged to be appropriate by relevant stakeholders (public, professional and political) [27]. Side effects/safety refers to whether the intervention has unwanted side effects or unintended consequences that need to be considered [27]. Equity refers to the extent to which an intervention may reduce or increase the disparities in standards of living, wellbeing or health between different sectors of society [27]. When there were different opinions on the selection of the intervention function, they were determined through group discussion. The research group members were PhD candidates or holders in nursing, had research interests in chronic disease nursing and had learning experience in behavioral science, which contributed to making this research more scientific and rigorous.

#### Step 6: Identifying policy categories

The step is to consider what policies would assist in the implementation of the identified intervention functions in Step 5. Policy categories included communication/marketing, guidelines, fiscal measures, regulation, legislation, environmental/social planning and service provision, which were determined using the APEASE criteria [27, 44]. Similarly, inconsistencies were resolved through group discussions.

### Stage 3: Identifying content and implementation options

#### Step7: Identifying BCTs

We identified BCTs as intervention strategies for promoting the desired behavior. Using APEASE criteria, we selected the BCTs that were commonly used from the BCTTv1 for each IF [27, 30] by two researchers. Moreover, a comprehensive matrix was used to map the 59 BCTs to the TDF domains to identify any additional BCTs [27]. We resolved any disagreements within our research team through discussion.

#### Step8: Model of delivery

An intervention delivery model refers to the way in which it is administered [45]. Various delivery models must be considered before choosing the most appropriate one, including face-to-face, TV, apps, and cell phone message [27]. The modes of delivery for BCTs were assessed using the APEASE criterion [27]. In addition, the selection of delivery models could also consider similar research of physical activity interventions among people with metabolic syndrome. Inconsistencies were resolved by the research team through discussions.

### Expert consultation

After all steps were completed, the key findings from each stage were synthesized. The intervention content and format were sent to 12 experts with different academic backgrounds through email, including advanced nursing practitioners, behavioral science experts, management scientists, and general physicians. All experts reviewed the intervention materials independently, and gave their feedback and comments (received via email after two weeks). We thoroughly reviewed and discussed each feedback and then revised the intervention content and format accordingly.

### Ethical consideration

The Helsinki Declaration was complied with. The participating hospitals’ ethics committees approved this study (grant no. 20210220–32). All the participants signed free and informed consent forms prior to starting the research. Participants were informed that their data were confidential.

## Results

### Step 1: Define the problem in behavioral terms

Physical activity was one of primary interventions in the management of metabolic syndrome [46]. Several studies summarized that adherence to physical activity recommendations, such as moderate physical activity of at least 150 min per week, vigorous activity of at least 75 min per week, or a combination of both, and total leisure-time energy expenditure of over 300 metabolic equivalents of task (MET)·min/day, was not adequate among adults with metabolic syndrome [13, 16–18]. Physical inactivity was associated with an increased risk of serious complications while regular physical activity led to increased energy consumption and was related to reducing the risk of metabolic syndrome [47]. Therefore, we defined the problem as the inadequate adherence to physical activity recommendations.

### Step 2: Select target behavior

Several studies proposed the standards of physical activity for individuals with metabolic syndrome, including type, time and frequency of physical activity [4, 48, 49]. Two evidence recommended a minimum of 30 min of moderate-intensive physical activity at least five days a week for individuals with metabolic syndrome [50, 51]. An international panel recommended the standard of daily physical activity for metabolic syndrome individuals was 30 ~ 60 min [4]. Pattyn et al., [52] presented that at least 40 min of aerobic training at least twice a week was effective on cardiovascular risk factors related to the metabolic syndrome. Among these potential target behaviors, we intended to choose the behavior that met the four rating criteria [27] including (i) how much of an impact changing the behavior will have on the desired outcome, (ii) how likely it is that the behavior can be changed, (iii) how likely it is that the behavior will have a positive or negative impact on other, related behaviors, and (iv) how easy it will be to measure the behavior [27] as the target behavior. Furthermore, the formation of habits is crucial for adopting a new behavior, which takes two to eight months to accomplish [53]. Based on the recommendations of physical activity from existing literature, the four rating criteria, and the time of habit formation, achieving a minimum of 30 min of moderate-intensive physical activity at least five days a week for 24 weeks was selected as the target behavior.

### Step 3: Specify target behavior

The specification of the target behavior is detailed in Table 2.

**Table 2:** Specifying the target behavior

Target behavior	Physical activity adherence
Who needs to perform the behavior?	People with metabolic syndrome
When will they do it?	When convenient to the persons with metabolic syndrome
Where will they do it?	At home and outside of the home
How often will they do it?	At least 30 min of moderate-intensive physical activity a minimum of 5 days aweek for 24 weeks
With whom will they do it?	Individual or group

### Step 4: What needs to change?

We employed the COM-B model and the TDF to perform a behavioral diagnosis among 28 individuals with metabolic syndrome. Tables 3 and 4 present the sample demographics and the results of behavioral diagnosis, separately. Overall, 33 themes were identified through in-depth interviews with people with metabolic syndrome in our study (see Table 4). The following identified barriers need to be changed: perceived poor knowledge about the diagnosis of metabolic syndrome; absent knowledge about regular physical activity; lacking self-monitoring; fearing that physical activity would aggravate conditions; absence of physical activity skills; lacking time; lacking equipment and venue; perceived poor physical activity atmosphere; the influence of weather; perceiving physical activity as unimportant; not perceiving benefits of physical activity; low intention; having intention but lacking confidence; no goals; and being influenced by negative emotions.

**Table 3:** Demographics of the sample (*n* = 28)

Variable		n (%)	Mean (SD)
Sex	Male	21 (75.0)	
	Female	7 (25.0)	
Age			50.46 ± 6.88
Residence	City	20 (71.4)	
	Town	4 (14.3)	
	Countryside	4 (14.3)	
Religion	Yes	5(17.9)	
	No	23 (82.1)	
Education level	≤ Middle school education	18 (64.3)	
	High school education or technical secondary school	4 (14.3)	
	Junior college	4 (14.3)	
	≥ University education	2 (7.1)	
Occupation	Leaders of administrative agencies, enterprises and institutions	4 (14.3)	
	Staff	3 (10.7)	
	Businessmen	8 (28.6)	
	Workers	1 (3.6)	
	Freelancers	8 (28.6)	
	Housewife	1 (3.6)	
	Others	3 (10.7)	
Co-residents	Parents	4 (14.3)	
	Son/daughter	13 (46.4)	
	Spouse	28 (100.0)

**Table 4:** Behavioral analysis based on the TDF and the COM‐B model

COM-B	TDF	Sub-theme	Quote	Barrier or facilitator
Psychological capability	Knowledge	Perceived limited knowledge about the diagnosis of metabolic syndrome	I have heard of high blood pressure, high blood glucose, and hyperlipidemia. But I am unfamiliar with metabolic syndrome. (P7)	Barrier
			Does it mean poor metabolism? I don’t know much about metabolic syndrome. (P14)	
		Absent knowledge about regular physical activity	I have no idea what regular physical activity is. And I don’t know how long it takes to do physical activity. (P15)	Barrier
			For me, regular exercise is to do what you can. I'm not sure if what I say is correct. (P16)	
			I don’t want to exercise. My mother has been suffering from diabetes for 30 to 40 years. She took medicine and received insulin treatment. She is in good health. (P19)	
			I can’t ensure that my physical activity is regular. Because I don’t know what regular physical activity is. (P6)	
	Behavior regulation	Lacking self-control	I do not have the habit of recording physical activity. (P6)	Barrier
	Memory, attention and decision process	Work driven	The process of work is also a process of physical activity. I sweat a lot at work. (P21)	Facilitator
		Hobby driven	I like dancing. I danced for 2 h a day for a month and lost 5 kg. (P13)	Facilitator
		The severity of metabolic syndrome	I don’t want to exercise because I'm too lazy. I want to sleep or play mahjong when I'm full. The metabolic syndrome is not severe. (P19)	Facilitator
			If the metabolic syndrome is severe, I will exercise. If not, I will not maintain regular exercise. (P2)	
		Pursuing perfection	I have been running for a year since July last year, and basically no one reminded me to exercise. This is related to my character of pursuing perfection. (P5)	Facilitator
Physical capability	Skills	Fearing that physical activity would aggravate conditions	I walk a little longer, and my knees won’t work. I originally liked skipping rope, because of my knees, I stopped skipping rope. (P2)	Barrier
			My lumbar muscle is strained, so I can’t do strenuous exercise. (P8)	
		Absence of physical activity skills	I have not received any physical activity-related training. I signed up for the gym, but never went to it once. (P3)	Barrier
			No one told me how to exercise. I exercise with my own experience. (P1)	
Social opportunity	Social influences	Social support is necessary	My wife reminded me to exercise regularly. It would be better to have someone remind. (P3)	Facilitator
			I rarely exercise by myself and don’t want to go at all. It needs at least two people to work out together. (P24)	
			I really need my wife’s supervision. My wife always reminds me to exercise, which motivates me to exercise. (P9)	
Physical opportunity	Environmental context and resources	Lacking time	I have to accompany my clients every night, and when I get home it’s very late, and I don’t have time to exercise. Therefore, work takes up exercise time. (P7)	Barrier
		Lacking equipment and venue	We used to like to play badminton in the factory. Later we changed our work	Barrier
			place, and there was no venue in the factory. Therefore, it is rare to play badminton nowadays. (P2)	
			I used to swim, but then I stopped swimming because the swimming pool was far away from us, which was inconvenient. (P23)	
		Lacking physical activity atmosphere	People in my hometown generally do not exercise until after the age of fifty, and rarely do exercises before the age of fifty. Everyone is working hard. (P17)	Barrier
		Good physical activity atmosphere	I used to feel boring to go swimming alone. There are two friends, they said they want to go swimming too. Then I have been insisting on going swimming. (P20)	Facilitator
			People of my dad’s generation are all exercising. I think I need also exercise. When I am in their age, I will be different from other people. (P4)	
		The influence of weather	I was supposed to jog for an hour every morning. I haven’t run for two months. The weather affects my physical activity, such as high temperature and rainy days. (P15)	Barrier
Reflective motivation	Social/professional role and identity	Physical activity is unimportant	I am fine. Exercise is optional in my life and it is not important. Some things are more important than exercise. (P3)	Barrier
		Work is more important than exercise	Exercise is not very important. It is a part of life, and the important thing is to make money. (P16)	Barrier
			It’s impossible (to exercise) now. After I retire, I will exercise. The important thing now is work. (P28)	
	Belief about capabilities	Sufficient confidence	I am very confident. I want to work until I am eighty. Because my child is still young, I have to work hard to support my family. (P8)	Facilitator
			Considering my health, it is not difficulty. It just needs me to get up earlier. (P16)	
	Beliefs about consequences	Good spirits	I feel refreshed after every workout. Therefore, physical activity will make people more energetic. (P17)	Facilitator
		Metabolic parameters and anthropometric indicators are improved	I used to jog every day. Through regular exercise, I lost 7 kg in 22 days. I can’t believe this fact. (P8)	Facilitator
			My blood glucose was high for a while. After a month of exercise, I don’t need to take blood glucose medicine anymore. (P5)	
		Physical fitness is improved	Physical fitness improves after exercise. If I do not exercise, I will pant when climbing stairs. (P7)	Facilitator
		Memory is improved	Exercise accelerates blood circulation, provides nutrition to brain cells, and then improves memory. (P10)	Facilitator
		Be beneficial to sleep	I didn't sleep well. Sleep quality was improved after exercise. Exercise helps sleep. (P27)	Facilitator
	Optimism	Confident	Deeply influenced by Chinese Yixue culture, I believe in mind therapy. If you believe that exercise has good results, it has good results. (P8)	Facilitator
			As everybody knows, physical activity will have many benefits. I understand the truth. (P10)	
		Not perceiving benefits of physical activity	I’ve been fat since I was a kid. I used to exercise and control my diet, but my weight has not improved significantly. (P6)	Barrier
			I may be predisposed to obesity. Exercise didn’t make me lose weight. Therefore, I don’t want to exercise anymore. (P14)	
	Intention	High intention	I am the oldest in my family. I have heavy responsibilities, and I can’t have any problems with my body. I will continue to maintain this (regular)exercise habit in the future. (P5)	Facilitator
			Don’t worry, I will definitely go on (dancing) for weight loss and health. (P13)	
		Have intention but lack of confidence	I plan to exercise regularly, but I feel like it is difficult to implement. I’m usually too busy. (P10)	Barrier
	Goals	No goals	I am a person who pursues freedom. Go as far as you want. No goals are set. (P11)	Barrier
		Keep healthy	The original intention of exercise is to reduce the waist circumference. Also, I want to live longer. Because my dad passed away in his 60 s. (P4)	Facilitator
			Only my own body is my own. I will be responsible for my own body. I must get my body well which is the greatest wealth. (P20)	
			I run five kilometers a day more often. I do it for health, not for running. (P5)	
Automatic motivation	Reinforcement	Yearning for knowledge and sunshine	When I am eager for knowledge and longing for sunshine, I go to climb the mountain. (P8)	Facilitator
		Monitor weight	I monitor my weight every day to urge myself to exercise regularly. When I gain weight, I exercise more. When I lose weight, I exercise a little less. (P20)	Facilitator
		The requirements of children	Rewards don’t make me exercise, nothing makes me exercise. Unless the children ask me to walk with them, I will exercise. (P19)	Facilitator
			When others force me to exercise, I will exercise. Without coercion, it is impossible for me to exercise. (P12)	
	Emotion	Negative emotions affect physical activity	Exercise is not appealing to me when I'm in a bad mood and I just want to sleep. (P11)	Barrier

### Step 5: Identifying intervention functions

In the present study, seven out of the nine possible intervention functions were selected to tackle the identified barriers using APEASE criteria: education, enablement, training, environment restructuring, persuasion, modeling and incentivization. Restriction was not included because the study was not involved using the rules to improve physical activity adherence. Coercion was excluded as punishment or cost were not acceptable for people with metabolic syndrome.

### Step 6: Identifying policy categories

As our study was intended to develop a behavior change intervention and was not involved with changing policy on physical activity based on the interview results, we did not address these policy categories and skipped this step.

### Step7: Identifying BCTs

In our study, nineteen BCTs were identified based on the APEASE criteria, including: information about health consequences (5.1); prompts/cues (7.1); self-monitoring of behavior (2.3); goal setting (behavior) (1.1); demonstration of the behavior (6.1); instruction on how to perform a behavior (4.1); feedback on the behavior (2.2); behavioral practice/rehearsal (8.1); social support (practical) (3.2); restructuring the social environment (12.2); credible source (9.1); commitment (1.9); behavioral contract (1.8); goal setting (outcome) (1.3); action planning (1.4); review behavior goal(s) (1.5); reduce negative emotions (11.2); emotional consequences (5.6); and social support (emotional) (3.3) (see Table 5). Other BCTs were excluded because they were ineffective, unacceptable, impracticable, or too expensive. Specific reasons could be found in Table 5.

**Table 5:** Identification of the possible BCTs that could be used in the intervention

TDF domains	IF identified	BCTs identified	Does the BCT meet the APEASE criteria?
Knowledge	Education	Information about social and environmental consequences (5.3)	No, not practicable in this context, as the intervention does not focus on social and environmental consequences
		Information about health consequences (5.1)	Yes
		Feedback on behavior (2.2)	No, it may be ineffective based on previous physical activity intervention experience
		Feedback on outcome(s) of the behavior (2.7)	No, it may be ineffective based on previous physical activity intervention experience
		Prompts/cues (7.1)	No, it may be ineffective based on previous physical activity intervention experience
		Self-monitoring of behavior (2.3)	No, it may be ineffective based on previous physical activity intervention experience
		Biofeedback (2.6)	No, it may be ineffective based on previous physical activity intervention experience
		Antecedents (4.2)	No, it may be ineffective based on previous physical activity intervention experience
Behavior regulation	Education	Information about social and environmental consequences (5.3)	No, not practicable in this context, as the intervention does not focus on social and environmental consequences
		Information about health consequences (5.1)	Yes
		Feedback on behavior (2.2)	No, it may be ineffective based on previous physical activity intervention experience
		Feedback on outcome(s) of the behavior (2.7)	No, it may be ineffective based on previous physical activity intervention experience
		Prompts/cues (7.1)	Yes
		Self-monitoring of behavior (2.3)	No, it may be ineffective based on previous physical activity intervention experience
	Training	Demonstration of the behavior (6.1)	No, it may be ineffective based on previous physical activity intervention experience
		Instruction on how to perform a behavior (4.1)	No, it may be ineffective based on previous physical activity intervention experience
		Feedback on the behavior (2.2)	No, it may be ineffective based on previous physical activity intervention experience
		Feedback on outcome(s) of behavior (2.7)	No, it may be ineffective based on previous physical activity intervention experience
		Self-monitoring of behavior (2.3)	No, it may be ineffective based on previous physical activity intervention experience
		Behavioral practice/rehearsal (8.1)	No, it may be ineffective based on previous physical activity intervention experience
	Modelling	Demonstration of the behavior (6.1)	No, it may be ineffective based on previous physical activity intervention experience
	Enablement	Social support (practical) (3.2)	No, it may be ineffective based on previous physical activity intervention experience
		Goal setting (behavior) (1.1)	No, it may be ineffective based on previous physical activity intervention experience
		Goal setting (outcome) (1.3)	No, it may be ineffective based on previous physical activity intervention experience
		Adding objects to the environment (12.5)	No, it is expensive for intervention designers
		Problem solving (1.2)	No, it may be ineffective based on previous physical activity intervention experience
		Action planning (1.4)	No, it may be ineffective based on previous physical activity intervention experience
		Self-monitoring of behavior (2.3)	Yes
		Restructuring the physical environment (12.1)	No, it is expensive for intervention designers
		Review behavior goal(s) (1.5)	No, it may be ineffective based on previous physical activity intervention experience
		Review outcome goal(s) (1.7)	No, it may be ineffective based on previous physical activity intervention experience
		Self-monitoring of behavior (2.3)	No, it may be ineffective based on previous physical activity intervention experience
		Behavioral practice/rehearsal (8.1)	No, it may be ineffective based on previous physical activity intervention experience
	Environmental restructuring	Adding objects to the environment (12.5)	No, it is expensive for intervention designers
		Prompts/cues (7.1)	Yes
		Restructuring the physical environment (12.1)	No, it is expensive for intervention designers
	Enablement	Social support (practical) (3.2)	No, it may be ineffective based on previous physical activity intervention experience
		Goal setting (behavior) (1.1)	Yes
		Goal setting (outcome) (1.3)	No, it may be ineffective based on previous physical activity intervention experience
		Adding objects to the environment (12.5)	No, it is expensive for intervention designers
		Problem solving (1.2)	No, it may be ineffective based on previous physical activity intervention experience
		Action planning (1.4)	No, it may be ineffective based on previous physical activity intervention experience
		Self-monitoring of behavior (2.3)	No, it may be ineffective based on previous physical activity intervention experience
		Restructuring the physical environment (12.1)	No, it is expensive for intervention designers
		Review behavior goal(s) (1.5)	No, it may be ineffective based on previous physical activity intervention experience
		Review outcome goal(s) (1.7)	No, it may be ineffective based on previous physical activity intervention experience
Skills	Training	Demonstration of the behavior (6.1)	Yes
		Instruction on how to perform a behavior (4.1)	Yes
		Feedback on the behavior (2.2)	Yes
		Feedback on outcome(s) of behavior (2.7)	No, it may be ineffective based on previous physical activity intervention experience
		Self-monitoring of behavior (2.3)	No, it may be ineffective based on previous physical activity intervention experience
		Behavioral practice/rehearsal (8.1)	Yes
		Graded tasks (8.7)	No, it may be ineffective based on previous physical activity intervention experience
		Habit reversal (8.4)	No, it may be ineffective based on previous physical activity intervention experience
		Body changes (12.6)	No, it may be ineffective based on previous physical activity intervention experience
		Habit formation (8.3)	No, it may be ineffective based on previous physical activity intervention experience
Environmental context and resources	Training	Demonstration of the behavior (6.1)	No, it may be ineffective based on previous physical activity intervention experience
		Instruction on how to perform a behavior (4.1)	No, it may be ineffective based on previous physical activity intervention experience
		Feedback on the behavior (2.2)	No, it may be ineffective based on previous physical activity intervention experience
		Feedback on outcome(s) of behavior (2.7)	No, it may be ineffective based on previous physical activity intervention experience
		Self-monitoring of behavior (2.3)	No, it may be ineffective based on previous physical activity intervention experience
		Behavioral practice/rehearsal (8.1)	No, it may be ineffective based on previous physical activity intervention experience
	Environmental restructuring	Adding objects to the environment (12.5)	No, it is expensive for intervention designers
		Prompts/cues (7.1)	Yes
		Restructuring the physical environment (12.1)	No, it is expensive for intervention designers
	Enablement	Social support (practical) (3.2)	Yes
		Goal setting (behavior) (1.1)	Yes
		Goal setting (outcome) (1.3)	No, it may be ineffective based on previous physical activity intervention experience
		Adding objects to the environment (12.5)	No, it is expensive for intervention designers
		Problem solving (1.2)	No, it may be ineffective based on previous physical activity intervention experience
		Action planning (1.4)	No, it may be ineffective based on previous physical activity intervention experience
		Self-monitoring of behavior (2.3)	No, it may be ineffective based on previous physical activity intervention experience
		Restructuring the physical environment (12.1)	No, it is expensive for intervention designers
		Review behavior goal(s) (1.5)	No, it may be ineffective based on previous physical activity intervention experience
		Review outcome goal(s) (1.7)	No, it may be ineffective based on previous physical activity intervention experience
		Restructuring the physical environment (12.2)	No, it is expensive for intervention designers
		Discriminative (learned) cue (7.2)	No, it may be ineffective based on previous physical activity intervention experience
		Prompts / cues (7.1)	Yes
		Restructuring the social environment (12.2)	Yes
		Avoidance / changing exposure to cues for the behavior (12.3)	No, it may be ineffective based on previous physical activity intervention experience
Social/professional role and identity	Education	Information about social and environmental consequences (5.3)	No, not practicable in this context, as the intervention does not focus on social and environmental consequences
		Information about health consequences (5.1)	Yes
		Feedback on behavior (2.2)	No, it may be ineffective based on previous physical activity intervention experience
		Feedback on outcome(s) of the behavior (2.7)	No, it may be ineffective based on previous physical activity intervention experience
		Prompts/cues (7.1)	No, it may be ineffective based on previous physical activity intervention experience
		Self-monitoring of behavior (2.3)	No, it may be ineffective based on previous physical activity intervention experience
	Persuasion	Credible source (9.1)	No, it may be ineffective based on previous physical activity intervention experience
		Information about health consequences (5.1)	Yes
		Feedback on behavior (2.2)	No, it may be ineffective based on previous physical activity intervention experience
		Information about social and environmental consequences (5.3)	No, not practicable in this context, as the intervention does not focus on social and environmental consequences
		Feedback on outcome(s) of the behavior (2.7)	No, it may be ineffective based on previous physical activity intervention experience
	Modelling	Demonstration of the behavior (6.1)	No, it may be ineffective based on previous physical activity intervention experience
Optimism	Education	Information about social and environmental consequences (5.3)	No, not practicable in this context, as the intervention does not focus on social and environmental consequences
		Information about health consequences (5.1)	Yes
		Feedback on behavior (2.2)	No, it may be ineffective based on previous physical activity intervention experience
		Feedback on outcome(s) of the behavior (2.7)	No, it may be ineffective based on previous physical activity intervention experience
		Prompts/cues (7.1)	No, it may be ineffective based on previous physical activity intervention experience
		Self-monitoring of behavior (2.3)	No, it may be ineffective based on previous physical activity intervention experience
	Persuasion	Credible source (9.1)	Yes
		Information about social and environmental consequences (5.3)	No, not practicable in this context, as the intervention does not focus on social and environmental consequences
		Information about health consequences (5.1)	Yes
		Feedback on behavior (2.2)	No, it may be ineffective based on previous physical activity intervention experience
		Feedback on outcome(s) of the behavior (2.7)	No, it may be ineffective based on previous physical activity intervention experience
	Modelling	Demonstration of the behavior (6.1)	No, it may be ineffective based on previous physical activity intervention experience
	Enablement	Social support (practical) (3.2)	No, it may be ineffective based on previous physical activity intervention experience
		Goal setting (behavior) (1.1)	No, it may be ineffective based on previous physical activity intervention experience
		Goal setting (outcome) (1.3)	No, it may be ineffective based on previous physical activity intervention experience
		Adding objects to the environment (12.5)	No, it is expensive for intervention designers
		Problem solving (1.2)	No, it may be ineffective based on previous physical activity intervention experience
		Action planning (1.4)	No, it may be ineffective based on previous physical activity intervention experience
		Self-monitoring of behavior (2.3)	No, it may be ineffective based on previous physical activity intervention experience
		Restructuring the physical environment (12.1)	No, it is expensive for intervention designers
		Review behavior goal(s) (1.5)	No, it may be ineffective based on previous physical activity intervention experience
		Review outcome goal(s) (1.7)	No, it may be ineffective based on previous physical activity intervention experience
		Verbal persuasion to boost self-efficacy (15.1)	No, it may be ineffective based on previous physical activity intervention experience
Intention	Education	Information about social and environmental consequences (5.3)	No, not practicable in this context, as the intervention does not focus on social and environmental consequences
		Information about health consequences (5.1)	Yes
		Feedback on behavior (2.2)	No, it may be ineffective based on previous physical activity intervention experience
		Feedback on outcome(s) of the behavior (2.7)	No, it may be ineffective based on previous physical activity intervention experience
		Prompts/cues (7.1)	Yes
		Self-monitoring of behavior (2.3)	No, it may be ineffective based on previous physical activity intervention experience
	Persuasion	Credible source (9.1)	Yes
		Information about social and environmental consequences (5.3)	No, not practicable in this context, as the intervention does not focus on social and environmental consequences
		Information about health consequences (5.1)	Yes
		Feedback on behavior (2.2)	No, it may be ineffective based on previous physical activity intervention experience
		Feedback on outcome(s) of the behavior (2.7)	No, it may be ineffective based on previous physical activity experience
	Incentivization	Feedback on behavior (2.2)	Yes
		Feedback on outcome(s) of behavior (2.7)	No, it may be ineffective based on previous physical activity intervention experience
		Monitoring of behavior by others without evidence of feedback (2.5)	No, it may be ineffective based on previous physical activity intervention experience
		Monitoring outcome of behavior by others without evidence of feedback (2.1)	No, it may be ineffective based on previous physical activity intervention experience
		Self-monitoring of behavior (2.3)	Yes
	Modelling	Demonstration of the behavior (6.1)	Yes
		Commitment (1.9)	Yes
		Behavioral contract (1.8)	Yes
Goals	Education	Information about social and environmental consequences (5.3)	No, not practicable in this context, as the intervention does not focus on social and environmental consequences
		Information about health consequences (5.1)	Yes
		Feedback on behavior (2.2)	No, it may be ineffective based on previous physical activity intervention experience
		Feedback on outcome(s) of the behavior (2.7)	No, it may be ineffective based on previous physical activity intervention experience
		Prompts/cues (7.1)	No, it may be ineffective based on previous physical activity intervention experience
		Self-monitoring of behavior (2.3)	No, it may be ineffective based on previous physical activity intervention experience
	Persuasion	Credible source (9.1)	No, it may be ineffective based on previous physical activity intervention experience
		Information about social and environmental consequences (5.3)	No, not practicable in this context, as the intervention does not focus on social and environmental consequences
		Information about health consequences (5.1)	Yes
		Feedback on behavior (2.2)	No, it may be ineffective based on previous physical activity intervention experience
		Feedback on outcome(s) of the behavior (2.7)	No, it may be ineffective based on previous physical activity intervention experience
	Incentivization	Feedback on behavior (2.2)	No, it may be ineffective based on previous physical activity intervention experience
		Feedback on outcome(s) of behavior (2.7)	No, it may be ineffective based on previous physical activity intervention experience
		Monitoring of behavior by others without evidence of feedback (2.5)	No, it may be ineffective based on previous physical activity intervention experience
		Monitoring outcome of behavior by others without evidence of feedback (2.1)	No, it may be ineffective based on previous physical activity intervention experience
		Self-monitoring of behavior (2.3)	No, health care professionals are reluctant to remind individuals to monitor behavior through incentivization
	Modelling	Demonstration of the behavior (6.1)	Yes
	Enablement	Social support (practical) (3.2)	No, it may be ineffective based on previous physical activity intervention experience
		Goal setting (behavior) (1.1)	Yes
		Goal setting (outcome) (1.3)	Yes
		Adding objects to the environment (12.5)	No, it is expensive for intervention designers
		Problem solving (1.2)	No, it may be ineffective based on previous physical activity intervention experience
		Action planning (1.4)	Yes
		Self-monitoring of behavior (2.3)	No,
		Restructuring the physical environment (12.1)	No, it is expensive for intervention designers
		Review behavior goal(s) (1.5)	Yes
		Review outcome goal(s) (1.7)	No, it may be ineffective based on previous physical activity intervention experience
Emotion	Persuasion	Credible source (9.1)	No, it may be ineffective based on previous physical activity experience
		Information about social and environmental consequences (5.3)	No, not practicable in this context, as the intervention does not focus on social and environmental consequences
		Information about health consequences (5.1)	No, it may be ineffective based on previous physical activity intervention experience
		Feedback on behavior (2.2)	No, it may be ineffective based on previous physical activity intervention experience
		Feedback on outcome(s) of the behavior (2.7)	No, it may be ineffective based on previous physical activity intervention experience
	Incentivization	Feedback on behavior (2.2)	No, it may be ineffective based on previous physical activity intervention experience
		Feedback on outcome(s) of behavior (2.7)	No, it may be ineffective based on previous physical activity intervention experience
		Monitoring of behavior by others without evidence of feedback (2.5)	No, it may be ineffective based on previous physical activity intervention experience
		Monitoring outcome of behavior by others without evidence of feedback (2.1)	No, it may be ineffective based on previous physical activity intervention experience
		Self-monitoring of behavior (2.3)	No, it may be ineffective based on previous physical activity intervention experience
	Modelling	Demonstration of the behavior (6.1)	No, it may be ineffective based on previous physical activity intervention experience
	Enablement	Social support (practical) (3.2)	No, it may be ineffective based on previous physical activity intervention experience
		Goal setting (behavior) (1.1)	No, it may be ineffective based on previous physical activity intervention experience
		Goal setting (outcome) (1.3)	No, it may be ineffective based on previous physical activity intervention experience
		Adding objects to the environment (12.5)	No, it is expensive for intervention designers
		Problem solving (1.2)	No, it may be ineffective based on previous physical activity intervention experience
		Action planning (1.4)	No, it may be ineffective based on previous physical activity intervention experience
		Self-monitoring of behavior (2.3)	No, it may be ineffective based on previous physical activity intervention experience
		Restructuring the physical environment (12.1)	No, it is expensive for intervention designers
		Review behavior goal(s) (1.5)	No, it may be ineffective based on previous physical activity intervention experience
		Review outcome goal(s) (1.7)	No, it may be ineffective based on previous physical activity intervention experience
		Reduce negative emotions (11.2)	Yes
		Emotional consequences (5.6)	Yes
		Self-assessment of affective consequences (5.4)	No, it may be ineffective based on previous physical activity intervention experience
		Social support (emotional) (3.3)	Yes

### Step8: Model of delivery

Apps are increasingly showing great promise in increasing individual physical activity adherence [54, 55]. Especially during the COVID-19 pandemic, health related apps seem to be more able to meet individual health needs. Three systematic reviews summarized that the apps-based interventions were effective in increasing physical activity for longer than 3 months [54–56]. The results of systematic review and meta-analysis showed that the mobile app-assisted interventions effectively improved health outcomes, including weight, blood glucose and blood pressure [57, 58]. Additionally, the advantages of apps also include convenience and being inexpensive and automation, and they allow users to receive health services in any environment and at any time. Given these attractive features that met the APEASE criteria, researchers started to deliver physical activity interventions via apps [59, 60]. According to previous experience of physical activity interventions, the context of epidemic era, features of app, our research team chose app as model of delivery.

### Expert consultation

Table 6 presents the mapping of the COM-B, TDF, barriers, intervention functions, BCTs, and potential intervention content. The main components constituted the intervention, including via the app (I) providing information on published research on the definition, negatives and physical activity-related knowledge of metabolic syndrome; (II) setting up reminders for individuals to record the type, time, frequency and (or) intensity of physical activity; (III) setting goals of physical activity; (IV) providing observable examples of individuals who perform physical activity properly; (V) providing video instruction on how to perform physical activity; (VI) providing suggestions on how to perform regular physical activity; (VII) presenting a speech from health care professionals to emphasize the benefits of physical activity for individuals with metabolic syndrome; (VIII) advising on the use of stress management skills, such as listening to music. Experts’ comments regarding the intervention content and format included:

-
  “Encourage individuals to rehearse physical activity properly via the app.” should be revised as “repeat the physical activity according to your physical condition until you master it”.
-
  “Inform the person of physical activity data” should be revised as “Inform the person of physical activity data and provide guidance”.
-
  “Follow and record various experiences of successfully maintaining regular exercise” should be added.
-
  “Record weight and WC every day” should be added.
-
  “Provide illustrations of the energy expenditure of physical activity” should be added.
-
  “Share own physical activity status with others via the app” should be added.
-
  “Exercise” should be modified to “physical activity”.
-
  “Set a weight loss goal as an outcome of regular physical activity.” should be revised as “Set a goal as an outcome of regular physical activity”.
-
  “Set up reminders for individuals to record the type, time, frequency and (or) intensity of physical activity via the app.” should be revised as “At 21:00 every night, remind the individual to record physical activity status via the app”.
-
  “Establish a contract with the individual to make sure to take regular physical activity via the app” may be not applicable in China.

**Table 6:** The intervention content identified based on BCTs

COM-B	TDF domains	Sub-theme/Barriers	Intervention functions identified	BCTs identified	Intervention content and format
Psychological capability	Knowledge	Perceived poor knowledge about the diagnose of metabolic syndrome	Education	Information about health consequences (5.1)	Provide information on published research on the definition, negatives, and physical activity-related knowledge of metabolic syndrome via the app
		Absent knowledge about regular physical activity			
	Behavior regulation	Lack of self-monitoring	Education	Information about health consequences (5.1)	Provide information on published research on the negatives and physical activity-related knowledge of metabolic syndrome via the app
				Prompts/cues (7.1)	Set up reminders for individuals to record the type, time, frequency, and (or) intensity of physical activity via the app
			Enablement	Self-monitoring of behavior (2.3)	Record the type, time, frequency, and (or) intensity of physical activity via the app
Physical capability	Skills	Fear of physical activity aggravating conditions	Training	Demonstration of the behavior (6.1)	Provide observable examples of individuals who perform physical activity properly via the app
		Absence of physical activity skills		Instruction on how to perform a behavior (4.1)	Provide instruction on how to perform physical activity via the app
				Feedback on the behavior (2.2)	Tell the person what they need to improve during physical activity via the app
				Behavioral practice/rehearsal (8.1)	Encourage individuals to rehearse physical activity properly via the app
Physical opportunity	Environmental context and resources	Lack of time	Environmental restructuring	Prompts/cues (7.1)	Set up reminders for individuals to perform physical activity via the app
		Lack of equipment and venue	Enablement	Social support (practical) (3.2)	Provide suggestions on how to perform regular physical activity (e.g., suggest users reduce time of entertainment to
					exercise; perform physical activity with devices and venues; suggest users exercise indoors on rainy days) via the app
		Perceived lack of physical activity atmosphere		Goal setting (behavior) (1.1)	Set goals of physical activity via the app
		The influence of weather		Restructuring the social environment (12.2)	Suggest users make friends with people who enjoy physical activity via the app
				Prompts/cues (7.1)	Set up reminders for individuals to perform physical activity via the app
Reflective motivation	Social/professional role and identity	Physical activity is unimportant	Education	Information about health consequences (5.1)	Provide information on published research on the definition, negatives, and physical activity-related knowledge of metabolic syndrome via the app
			Persuasion	Information about health consequences (5.1)	Introducing the negatives and physical activity-related knowledge of metabolic syndrome via the app
	Optimism	Not perceiving benefits of physical activity	Education	Information about health consequences (5.1)	Introducing the negatives and physical activity-related knowledge of metabolic syndrome via the app
			Persuasion	Credible source (9.1)	Present a speech from health care professionals to emphasize the benefits of physical activity for individuals with metabolic syndrome via the app
				Information about health consequences (5.1)	Introducing orally the negatives and physical activity-related knowledge of metabolic syndrome via the app
	Intention	Low intention	Education	Information about health consequences (5.1)	Provide information on published research on the definition, negatives, and physical activity-related knowledge of metabolic syndrome via the app
		Have intention but lack of confidence		Prompts/cues (7.1)	Set up reminders for individuals to perform physical activity via the app
			Persuasion	Credible source (9.1)	Present a speech from health care professionals to emphasize the importance of physical activity via the app
				Information about health consequences (5.1)	Provide information on published research on the definition, negatives, and physical activity-related knowledge of metabolic syndrome via the app
			Incentivization	Feedback on behavior (2.2)	Inform the person of physical activity data, such as how many steps they walk each day via the app
				Self-monitoring of behavior (2.3)	Record the type, time, frequency, and (or) intensity of physical activity via the app
			Modelling	Demonstration of the behavior (6.1)	Provide observable examples of individuals who enjoy physical activity via the app
				Commitment (1.9)	Ask the individuals to use the words, such as “strongly”, “committed” or “high priority” “I will” to affirm or reaffirm a strong commitment to start, continue, or restart the attempt to perform regular physical activity via the app
				Behavioral contract (1.8)	Establish a contract with the individual to make sure to take regular physical activity via the app
	Goals	No goals	Education	Information about health consequences (5.1)	Provide information on published research on the definition, negatives, and physical activity-related knowledge of metabolic syndrome via the app
			Persuasion	Information about health consequences (5.1)	Introducing the definition, negatives, and physical activity-related knowledge of metabolic syndrome via the app
			Modelling	Demonstration of the behavior (6.1)	Provide observable examples of individuals who enjoy physical activity via the app
			Enablement	Goal setting (behavior) (1.1)	Set goal of physical activity via the app
				Goal setting (outcome) (1.3)	Set a weight loss goal as an outcome of regular physical activity via the app
				Action planning (1.4)	Prepare a schedule to perform physical activity at a certain time and on certain days of the week via the app
				Review behavior goal(s) (1.5)	Examine if own performance corresponds to agreed-upon goals, and adjust future behavioral goals accordingly via the app
Automatic motivation	Emotion	Negative emotion affects physical activity	Enablement	Reduce negative emotions (11.2)	Providing advice on how to manage stress, such as listening to music via the app
				Emotional consequences (5.6)	Introduce the benefits of physical activity, such as relieving negative emotions via the app
				Social support (emotional) (3.3)	Consider taking a partner or friend with self to perform regular physical activity via the app

Experts suggested that in addition to the app, it is recommended to add other forms of intervention, such as mobile phone calls or telephone calls.

## Discussion

The present study outlines a rigorous theory-based method to develop a complex intervention to increase physical activity adherence among people with metabolic syndrome in China. To date, this paper is the first to use the BCW in this context and population. The findings demonstrated that changing physical activity behaviors needs to consider various factors, including the capability, opportunity, and motivation of individuals and choose suitable BCTs to support identified intervention functions. Our study provided the opportunity for health care professionals to better understand multifactorial influences based on theory on physical activity adherence among individuals with metabolic syndrome. It also extended the use of the BCW framework for developing physical activity interventions to target behavioral barriers to physical activity adherence in this population.

To improve compliance with physical activity, interventions should leverage facilitators and overcome barriers. The study identified seven intervention functions to mainly tackle fifteen barriers according to the APEASE criteria. Furthermore, nineteen BCTs were selected to assist in the delivery of seven intervention functions and were then translated into potential intervention content.

We found that nearly all participants lacked knowledge about the diagnosis of metabolic syndrome from interview results. These findings were in line with a previous study that showed poor knowledge about the definition and diagnosis of metabolic syndrome among adults with metabolic syndrome [61]. The phenomenon may be attributable to the fact that metabolic syndrome is underdiagnosed and undertreated due to it being largely asymptomatic [62]. Thus, specific health education on the definition of metabolic syndrome should be provided. Additionally, due to the participants' inadequate knowledge about physical activity, it is essential to educate them on its benefits and teach them how to perform it [34]. Furthermore, lacking self-monitoring of physical activity behavior was a barrier to physical activity. As a component of the BCTs, self-monitoring was conducive to motivating individuals to engage in physical activities [63]. Hence, enabling individuals to write physical activity diaries and use pedometers may increase adherence to physical activity.

The present study also found that the adherence to physical activity may be increased through the restructuring social and physical environment. In the present study, suggesting users make friends with people who like physical activity was a way to restructure the social environment, through which, individuals were more likely to regard exercise as the new “normal” [64], thereby enhancing the enthusiasm for performing physical activity. In addition, in accordance with our results, the physical environmental barriers to undertaking physical activity were time, weather, and facilities among middle-aged and older adults [65, 66]. Hence, it is imperative to restructure the physical environment, for example, arranging time reasonably to help themselves integrate physical activity into their schedule and participating in physical activity with equipment and venues.

Our behavior analysis presented that some respondents had the intention to participate in physical activity but lacked confidence, which was important for successful physical activity adherence [14]. According to Zelle et al. [67], it was an effective approach to increase self‐efficacy through persuading individuals that they had the ability to conduct a behavior, and encouraging them to do so. For persons who did not perceiving benefits of physical activity, offering an opportunity to let them experience small accomplishments in their performance was also conducive to improving self-efficacy [68]. However, some participants had no intention to conduct physical activity, had no goals when undertaking physical activity and regarded it as unimportant. Lacking adequate understanding of the metabolic syndrome, individuals could be unaware of the presentation of the metabolic syndrome and their complication risks. Thus, health education targeting metabolic syndrome including the disease risk, the benefits of physical activity and setting goals should be enhanced by healthcare professionals. In addition, negative emotions affect physical activity. This aligns with the literature, which showed that people with anxiety and/or depression were characterized by sedentary and low levels of physical activity completion recommendations [69]. Using stress management skills, such as listening to music, could reduce stress [70] and then help enable individuals to do more physical activity [71].

In terms of the intervention functions, seven intervention functions, including education, persuasion, training, modelling, incentivization, environmental restructuring, and enablement, were identified as relevant for physical activity intervention. The study by Truelove et al., [59] selected six intervention functions (education, persuasion, incentivization, training, environment restructuring, and enablement) in a physical activity app intervention design, which was in accordance with our work. Moreover, this study identified nineteen potential BCTs from the qualitative data. These results are similar to the two studies [34, 35] in which twenty-one BCTs and fourteen BCTs were identified to promote physical activity behavior, separately. Most studies often used BCTs combinations to promote physical activity. Future research could examine which particular BCT or combinations of BCTs are most effective in changing the physical activity behavior among people with metabolic syndrome via the app.

### Limitations

Although the study employed a strong theory to explore the influence mechanisms of action, our results must be interpreted cautiously with some limitations. First, all participants in the behavior analysis step were from Zhejiang Province in China. Therefore, the findings may be only applicable to people living with metabolic syndrome in China. Second, theme saturation was achieved, but given the disadvantages of theme saturation, we should interpret our findings with caution. Third, we did not identify all barriers and facilitators for increasing adherence to physical activity as we did not invite all key stakeholders in the present study, such as health care professionals, individuals’ relatives or friends. Fourth, it is essential to acknowledge the subjectivity of this analysis, as with many qualitative results, as well as concerns over external validity caused by a relatively small sample size. Fifth, a steering group was consulted only at certain steps, not all steps, which may lead to imperfect intervention design. Sixth, when selecting intervention functions and BCTs according to APEASE criteria, our research team did not also invite a multidisciplinary team. As a result, subjectivity existed. Seventh, our study did not focus primarily on changing policies, so we did not analyze policy categories. In future research, policy categories analysis is needed to help identify service provision, guidelines, environmental/social planning, and regulations for promoting behavioral change. Finally, when we applied the BCW, the intervention design process needs longer time. Therefore, efficiency of use was a potential problem.

### Future research

With the guidance of the BCW framework, we have identified core ingredients that can be incorporated into the intervention design to facilitate adherence to physical activity. Subsequently, we will invite software engineers to design the app features based on the intervention content. In the future, a randomized controlled trial (RCT) evaluating the feasibility, effectiveness, and acceptability of the physical activity program will be needed. If effective, health care professionals could provide the intervention content for adults with metabolic syndrome to target barriers to physical activity and ultimately improve their health outcomes. Additionally, the intervention program could also be adapted for use in other health conditions where physical activity adherence needs to be addressed.

## Conclusions

This study used a systematic approach to develop an intervention underpinned by the BCW theory to increase physical activity in adults living with metabolic syndrome in China, which may in turn improve the health outcomes for these individuals and reduce medical burden and economic burden. This study has identified nineteen BCTs, which can be used as active ingredients in intervention program of targeting behaviors determinants. Future studies should focus on whether the targeted intervention program enhances physical activity adherence and is accepted by metabolic syndrome individuals, ultimately to promote positive behavior change and improve health outcomes of individuals.  

## Data Availability

The datasets generated and/or analyzed during the current study are not publicly available due to the risk of breaking anonymity being too high but are available from the corresponding author on reasonable request.

## Abbreviations

APEASE: Affordability, Practicability, Effectiveness and Cost-effectiveness, Acceptability, Side effects/safety and Equity
BCTs: Behavior change techniques
BCTTv1: Version 1 of the BCT taxonomy
BCW: Behavior Change Wheel
CINAHL: Cumulative Index of Nursing and Allied Health Literature
COM-B: Capability, Opportunity, Motivation – Behavior
DBP: Diastolic blood pressure
FBG: Fasting blood glucose
HDL-C: Low high-density lipoprotein cholesterol
RCTs: Randomized controlled trails
SBP: Systolic blood pressure
TDF: Theoretical Domains Framework
TG: Triglyceride
SRQR: Standards for Reporting Qualitative Research
WC: Waist circumference
